# Phylogenetic and Complementation Analysis of a Single-Stranded DNA Binding Protein Family from Lactococcal Phages Indicates a Non-Bacterial Origin

**DOI:** 10.1371/journal.pone.0026942

**Published:** 2011-11-04

**Authors:** Agnieszka K. Szczepankowska, Eric Prestel, Mahendra Mariadassou, Jacek K. Bardowski, Elena Bidnenko

**Affiliations:** 1 Institut Micalis, UMR1319, INRA, Jouy-en-Josas, France; 2 Department of Microbial Biochemistry, Institute of Biochemistry and Biophysics PAS, Warsaw, Poland; 3 Mathématique, Informatique et Génome, UR1077, INRA, Jouy-en-Josas, France; J. Craig Venter Institute, United States of America

## Abstract

**Background:**

The single-stranded-nucleic acid binding (SSB) protein superfamily includes proteins encoded by different organisms from Bacteria and their phages to Eukaryotes. SSB proteins share common structural characteristics and have been suggested to descend from an ancestor polypeptide. However, as other proteins involved in DNA replication, bacterial SSB proteins are clearly different from those found in Archaea and Eukaryotes. It was proposed that the corresponding genes in the phage genomes were transferred from the bacterial hosts. Recently new SSB proteins encoded by the virulent lactococcal bacteriophages (Orf14_bIL67_-like proteins) have been identified and characterized structurally and biochemically.

**Methodology/Principal Findings:**

This study focused on the determination of phylogenetic relationships between Orf14_bIL67_-like proteins and other SSBs. We have performed a large scale phylogenetic analysis and pairwise sequence comparisons of SSB proteins from different phyla. The results show that, in remarkable contrast to other phage SSBs, the Orf14_bIL67_–like proteins form a distinct, self-contained and well supported phylogenetic group connected to the archaeal SSBs. Functional studies demonstrated that, despite the structural and amino acid sequence differences from bacterial SSBs, Orf14_bIL67_ protein complements the conditional lethal *ssb-1* mutation of *Escherichia coli*.

**Conclusions/Significance:**

Here we identified for the first time a group of phages encoded SSBs which are clearly distinct from their bacterial counterparts. All methods supported the recognition of these phage proteins as a new family within the SSB superfamily. Our findings suggest that unlike other phages, the virulent lactococcal phages carry *ssb* genes that were not acquired from their hosts, but transferred from an archaeal genome. This represents a unique example of a horizontal gene transfer between Archaea and bacterial phages.

## Introduction

Single-strand-nucleic acid binding (SSB) proteins have been identified in all domains of life (Eukaryotes, Archaea, Bacteria) and viruses. Proteins belonging to the SSB superfamily are involved in diverse aspects of DNA metabolism [1], [2]. They assure elimination of secondary structures and protection of ssDNA from nucleolitic degradation, but also coordinate the action of different proteins involved in genome maintenance machinery [3]–[5]. Despite their amino acid sequence diversity, SSB proteins share common characteristics at the structural level. The most important is the presence of a specific ssDNA-binding OB-fold (Oligonucleotide/Oligosaccharide Binding fold) domain, which constitutes the main core of the protein [6]. The OB-fold consists of a mixed β-barrel structure which, in contrast to the additional non-conserved structures present in the ssDNA-binding proteins, shows high structural stability and evolutionary conservation [7], [8]. Amino acids in the OB-fold are directly involved in binding of the protein monomer to DNA [9]–[11]. Most bacterial SSBs are homotetramers with a single OB-fold in each polypeptide [12], [13]. Eukaryotic ssDNA-binding replication protein A (RPA) is composed of three subunits (RPA70, RPA32 and RPA14) that form a heterotrimeric SSB with six OB-folds [2], [14], [15]. SSBs encoded by Euryarchaeotes are more similar to their eukaryotic analogues than to eubacterial SSBs. Some Crenarchaeotes harbor a single gene encoding a single OB-fold SSB, structurally similar to bacterial SSBs [16]–[19]. Genes encoding for SSB proteins have also been found in numerous bacteriophage (phage) genomes. The characterized phage SSBs include those of coli-phages T7, N4 and P1, as well as those encoded by other phages, e.g. *Bacillus* Ø29-like phages [20]–[25]. Phage-encoded SSB proteins have various roles, depending on the phage life cycle. For some phages, specific SSBs are involved in DNA replication and, in some cases are essential for phage development [20], [25]–[26]. Phylogenetic analysis of a number of bacterial and phage SSB proteins indicates that there have been frequent horizontal transfers from bacterial hosts to the genomes of their phages [27]. The bacterial origin of the phage *ssb* genes is easily recognized despite the high evolution rate of phages [24], [27]. Moreover, some phage SSBs are interchangeable with their bacterial analogs *in vivo* and *in vitro* [23], [28].

We previously characterized a novel SSB protein (Orf14_bIL67_) encoded by the virulent phage bIL67 infecting *Lactococcus lactis*, a Gram-positive bacterium used by the dairy industry [29]. Three-dimensional modeling *in silico* revealed a putative OB-fold domain in the Orf14_bIL67_ structure. The ssDNA-binding character of the Orf14_bIL67_ protein and the role of particular amino acid residues in the OB-fold domain were confirmed through electrophoretic mobility shift assays. We proposed that Orf14_bIL67_ is a member of a new SSB family comprising homologous gene products identified in virulent *Siphoviridae* lactococcal phages from 936- and c2-groups [29], [30]. This hypothesis was further supported by recent establishment of crystal structure of the second SSB protein from this group, Orf34_p2_, encoded by 936-like phage p2 [31].

Here we performed phylogenetic analyses which suggested that the Orf14_bIL67_-like proteins constitute a separate group on the basis of their evolutionary descent. In spite of their phylogenetic distance they are able to functionally complement a conditional *E. coli ssb-1* (ts) mutation.

## Results

### Cluster analysis of the SSB proteins

Proteins of the Orf14_bIL67_ SSB family, unlike SSBs encoded by other phages specific for *Lactobacillales*, show no sequence similarity to SSBs from their bacterial hosts [29], [31]. This raises a question about the relationships between Orf14_bIL67_-like proteins and other SSBs encoded by lactic acid bacteria and their phages.

We first built a dataset including 729 sequences of putative SSBs encoded by bacterial, archaeal, phage, eukaryotic nuclear and mitochondrial genomes (Material and Methods). The sequences from the SSB dataset were compared all-against-all with BLAST, to find clusters of similar sequences and visualize their sequence relationships, as implemented in the CLANS (CLuster ANalysis of Sequences) program [32]. CLANS allows classification of huge amount of unaligned protein sequences: highly similar sequences are clustered together and connected proportionally to their pairwise *p*-value (Materials and Methods).

**Figure 1:**
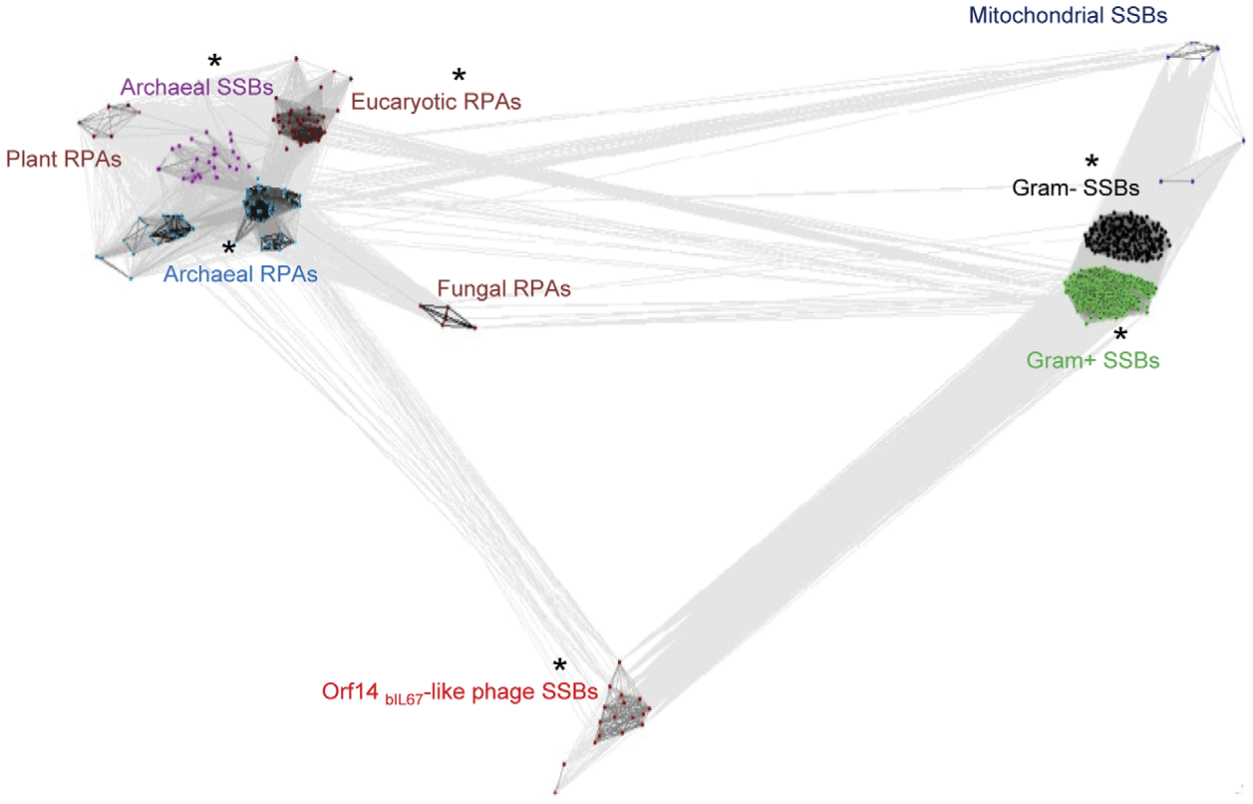
Cluster map of the ssDNA-binding protein superfamily. The complete sequence dataset for SSB proteins was clustered using CLANS, which classified all ssDNA-binding proteins into 10 subfamilies containing related proteins. CLANS runs BLAST on given sequences and clusters them in 3D according to their all-against-all pairwise similarity. A 2D representation was obtained by seeding sequences randomly in the arbitrary distance space. Protein subfamilies containing members with known structures are indicated with asterisk. In the network, each dot represents a single protein. Sequences of phages encoding Orf14_bIL67_ -like SSBs proteins are shown in red. Other colours: purple – Crenarchaea , blue – Euryarchaea, maroon – Eukaryotes, dark blue – mitochondria, black – Gram-negative bacteria, green – Gram-positive bacteria.

CLANS identified 10 well-defined families (clans) of ssDNA -binding proteins (Figure 1). These clans correspond to the SSB proteins encoded by Gram-positive and Gram-negative bacteria, mitochondrial SSBs, SSB and RPA proteins encoded by Archaea and RPA proteins encoded by eukaryotes respectively. Two clans of the bacterial SSBs (Gram-negative and Gram-positive bacteria) also include phage encoded SSB proteins and are the most compact ones indicating a high level of sequence conservation.

Interestingly, none of the previously identified Orf14_bIL67_ –like proteins were detected within the bacterial clans. Clustering revealed these proteins as a distinct well-defined clan with an evolutionary link to SSBs of Gram-positive bacteria and ssDNA-binding proteins of Archaea, as indicated by the relative positions of these clans. In addition to 11 previously identified Orf14_bIL67_ –like proteins encoded by lactococcal phages p2, bIL66M1, sk1, bIL170, jj50, P008, bIBB29, 712, c2, bIL67 and P335 (see Material and Methods for accession numbers) this clan includes 7 new members. These new members are encoded by virulent lactococcal phages SL4, SB13, SB14, CB19 and CB20, the lactic acid bacterium *Oenococcus oeni* AWRIB429 429 and prophage “2” of *L. lactis* subsp. *cremoris* SK11 (Materials and Methods). The gene encoding the putative SSB protein of *O. oeni* AWRIB429 429 maps to a 1382 bp DNA segment which is an integral part of the genome shotgun sequence. The DNA sequence along the entire length of this fragment shares up to 92% identity with DNA of lactococcal 936-like phages (Figure S1). So it is an insertion of the phage DNA representing a prophage-like element. Therefore, the new SSB clan proposed here consists exclusively of the proteins from lactococcal bacteriophages.

To assess whether the findings obtained by CLANS remain valid when using the sequences of characteristic ssDNA-binding OB-fold domains common in all SSB and RPA proteins, instead of complete protein sequences, we built a second dataset, containing only the OB-fold domains from the previous 729 proteins (Materials and Methods). The CLANS analysis of this trimmed dataset gives the same results as the analysis of complete sequences: the Orf14_bIL67_ OB-fold domain clan is again well separated from other OB-fold domain clans, and still connected to the bacterial and archaeal SSBs (Figure S2). Thus, CLANS analysis, performed on both full length proteins and conserved OB-fold domains only, provide evidence that Orf14_bIL67_-like proteins are clearly distinct from bacterial and archaeal SSBs, as well as from eukaryotic and archaeal RPA proteins.

### Phylogenetic analysis of the Orf14_bIL67_ SSB protein family

The recognition of the Orf14_bIL67_-like proteins as a separated SSB family, distinct from bacterial SSBs, but connected to archaeal SSBs, is intriguing. This prompted us to perform phylogenetic analyses of the Orf14_bIL67_ family to gain insight into the evolutionary links relating these proteins to other families of SSBs.

**Figure 2:**
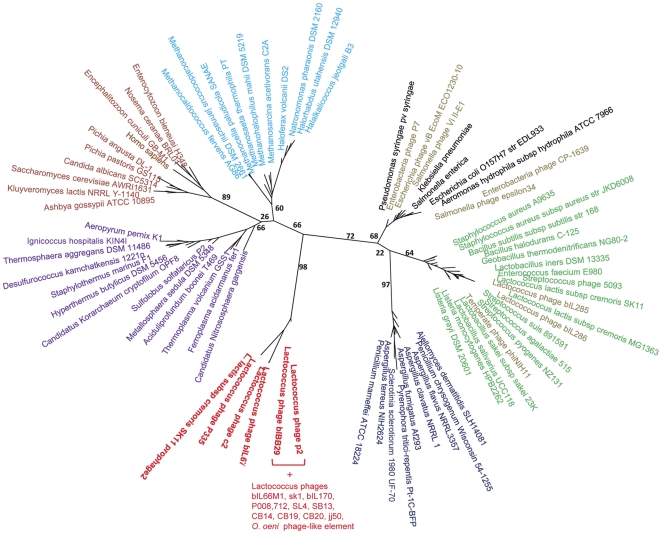
Unrooted Maximum Likelihood phylogenetic tree of the ssDNA-binding proteins. The SSB phylogeny was reconstructed using PhyML from multiple alignments that were generated using the “accurate” mode of T-coffee (Materials and Methods). Bootstrap support values are shown. Sequences of phages encoding Orf14_bIL67_ -like SSBs proteins are shown in red. Other colours: purple – Crenarchaea , blue – Euryarchaea, maroon – Eukaryotes, dark blue – mitochondria, black – Gram-negative bacteria, green – Gram-positive bacteria, and olive – phages.

A representative and balanced sample of 78 ssDNA-binding protein sequences from the 10 identified families was used to construct phylogenetic trees (Materials and Methods). The trees were built on OB-fold multiple sequence alignment using the Maximum Likelihood (ML) and Neighbour Joining (NJ) methods (Materials and Methods). The ML tree shows a clear separation of SSB proteins into several major lineages on the basis of their OB-fold domains alignment: Orf14_bIL67_-like SSBs, bacterial SSBs, archaeal SSBs, archeal RPAs and eukaryotic RPAs (Figure 2). These results are consistent with those obtained by CLANS (Figure 1 and Figure S2). All bacterial SSBs falls within the single branch, which as described earlier, is divided into two clades: Gram-positive and Gram-negative bacteria [33]. As expected, the mitochondrial SSBs fall within the same branch, in agreement with the apparent eubacterial origin of the mitochondrial genome [34]. The majority of the phage-encoded SSBs are present in one or other of the bacterial clade according to their host range and evolutional origin, as reported previously for some of them [24], [27].

As revealed by CLANS, Orf14_bIL67_-like proteins form a distant clade (Figure 2). They are well separated (bootstrap >95) from bacterial SSBs and affiliate to archaeal SSBs. The exact position of Orf14_bIL67_-like SSBs within archaeal proteins is not precise as the resolution of the archaeal/Orf14_bIL67_-like SSBs branching is poor (Figure 2). The Orf14_bIL67_-like SSBs in turn are divided in three groups. The largest monophyletic group is formed by 14 SSBs encoded by 936-like phages. The second and third groups are constituted by SSBs encoded by two c2-like lactococcal phages (c2 and bIL67) and two P335-like phages (*L. lactis* SK11 prophage “2” and phage P335) (Materials and Methods). It is remarkable, that all P335 phages (at least 28 genomes are available in public databases), except the two described above, encode SSB proteins homologous to the *L. lactis* SSB that explain their position within the bacterial clade designated by CLANS and phylogenetic analysis. Thus, the overwhelming majority (16 out of 18) of proteins forming the distinct Orf14_bIL67_-like SSB branch are encoded by virulent lactococcal phages from 936- and c2-like phage species.

Phylogenetic trees are notoriously difficult to infer from short divergent sequences, explaining the weak support of certain branches. It is thus necessary to check whether Orf14_bIL67_-like SSBs still stand out as a well supported clade when changing inference parameters. Our results show that the coherence and distinctive features of Orf14_bIL67_-like SSBs are remarkably robust: these proteins form a self-contained clade with high bootstrap values (>95), no matter what evolution model and/or number of rate categories are used (Materials and Methods). We checked for consistency that a similar topology with similar bootstrap values is found both in the NJ tree (Figure S3) and when using different alignment software (Material and Methods, Figure S4).

The separation of Orf14_bIL67_-like SSBs from the bacterial SSBs could be due to the classical phenomenon of long-branch attraction [35]. However, the longest branches in the tree are those leading to Orf14_bIL67_-like and to fungal SSBs (approximately 1.99 and 1.65), so we would expect the long-branch attraction to group Orf14_biL67_-like and fungal branches together, but they appear to be reasonably separated (with bootstrap values around 70%). Interestingly, the long-branch problem is mitigated for the tree reconstructed with known structure information (Figure 2 and Figure S4).

The terminal branches of the Orf14_biL67_-like SSB clade are short (<0.1). This is in agreement with the high level of amino acid (aa) conservation within the clade: up to 53% of aa similarity and 35% of aa identity between SSBs from 936 and c2 phage species and up to 97% of aa identity between SSBs from the same phage species. We searched for Orf14_bIL67_ homologous proteins using iterative sequence profile searches with the PSI-BLAST program, with inclusion thresholds of 0.005 (the default) and 0.05 (Materials and Methods). In both cases, convergence occurred after two iterations, and PSI-BLAST identified only 18 homologous proteins: exactly those found in the Orf14_bIL67_ family.

Thus, phylogenetic analyses strongly supports the results of CLANS pairwise sequence comparison and shows that the Orf14_bIL67_-like proteins form a family which is clearly distinct from bacterial, archaeal and eukaryotic ssDNA-binding proteins. Both methods stress relationships between Orf14_bIL67_-like and archaeal proteins.

### Expression of Orf14_bIL67_ improves the growth of the *E. coli* ssb-1 (ts) mutant

**Figure 3:**
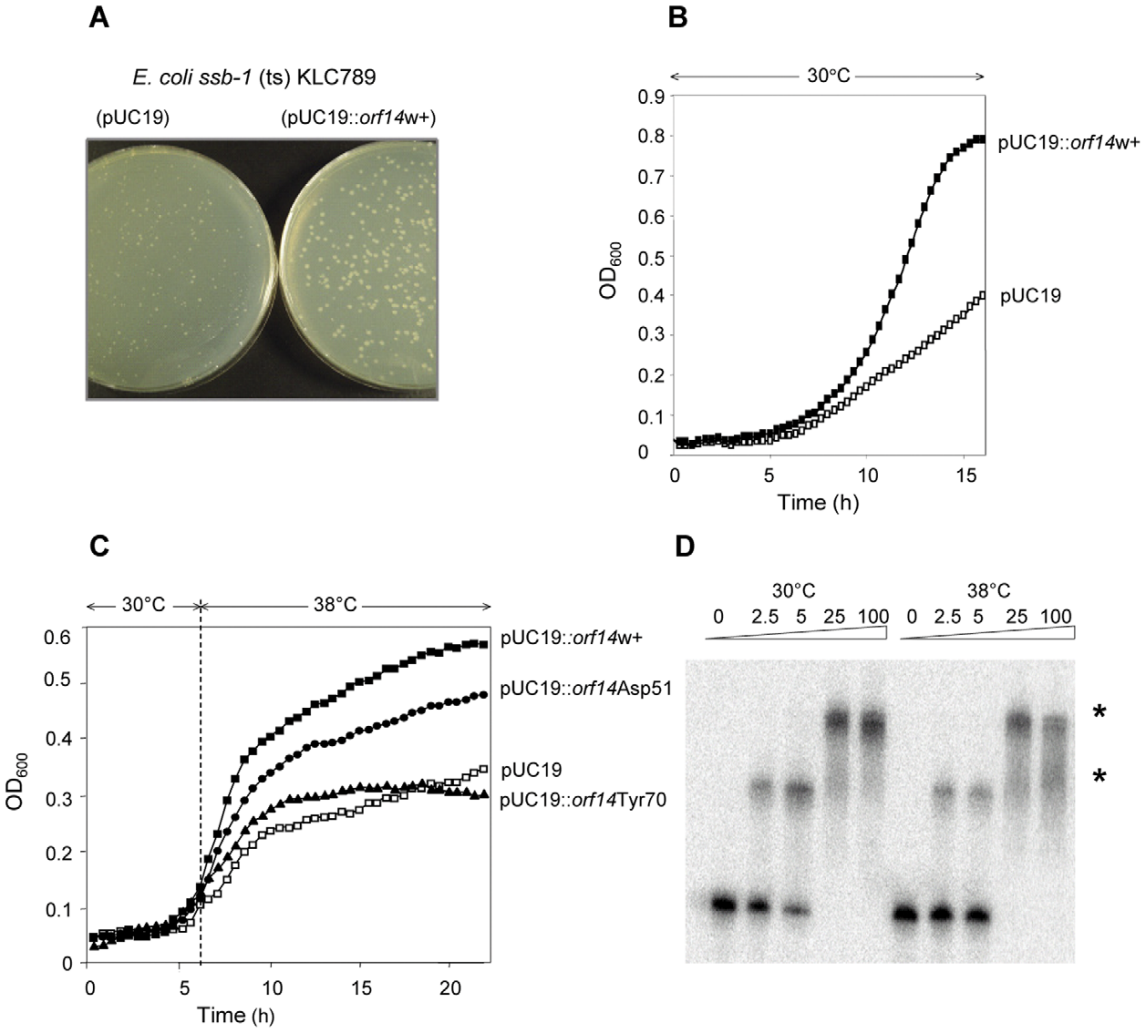
Rescue of the *E. coli ssb-1* mutant strain KLC789 by the *orf14_bIL67_*. **A:** Colony morphology of KLC789 (pUC19) and KLC789 (pUC19:*orf14_bIL67_*) strains on an LB agar plates in the presence of 0.25 mM of IPTG after 18 hours of incubation at 30°C. **B:** KLC789 (pUC19) (□) and KLC789 (pUC19:*orf14_bIL67_*) (▪) strains were grown in LB broth at 30°C in the presence of 0.25 mM of IPTG. **C:** KLC789 (pUC19) (□), KLC789 (pUC19:*orf14_bIL67_*) (▪), KLC789 (pUC19:*orf14_bIL67_*/D51A) (•) and KLC789 (pUC19:*orf14_bIL67_*/Y70A) (▴) strains were grown for 6 h at 30°C and then shifted to 38°C in a Bioscreen C apparatus (LabSystems), under continuous monitoring of OD_600_. The curves are generated from the means of four to six independent experiments; standard deviations were ≤10% (not shown). **D:** Binding of purified Orf14_bIL67_ to ssDNA at 30°C and 38°C. A series of concentrations (marked at the top) of the protein were incubated with 0.5 nM of an 80-mer oligonucleotide at two different temperatures (30°C or 38°C) prior to loading on the gel. Asterisks (*) mark the nucleoprotein complexes. The arrow indicates the position of free ssDNA.

The distinctive features of Orf14_bIL67_-like proteins raise questions about their functionality in bacterial cells. To examine whether Orf14_bIL67_ *in vivo* shares common properties with the representative bacterial *Eco*SSB protein we used a conditional *E. coli ssb-1* (ts) KLC789 mutant strain [36]. We tested whether the activity of Orf14_bIL67_ restored growth of the *ssb-1* mutant strain at non-permissive temperature. The *E. coli* KLC789 cells carrying the recombinant plasmid with the *orf14_bIL67_* gene cloned under the control of inducible promoter were grown at the permissive temperature of 30°C and at non-permissive temperature, 38°C (Materials and Methods). At 30°C, KLC789 (pUC19:*orf14_bIL67_*) cells formed colonies of normal morphology, whereas the KLC789 (pUC19) control strain formed small colonies with dry colony morphology (Figure 3A). Plating of the KLC789 (pUC19:*orf14_bIL67_*) culture at 38°C resulted in the formation of small colonies, whereas KLC789 (pUC19) did not grow at all (data not shown).

The second test was performed by growing *E. coli* KLC789 cells in LB broth either continuously at 30°C (Figure 3B) or by shifting the temperature to 38°C after 6 hours at 30°C (Figure 3B and Materials and Methods). The presence of the *orf14_bIL67_* gene *in trans* improved growth at 30°C (Figure 3B). The temperature shift from 30°C to 38°C resulted in growth defect of the *E. coli* KLC789 (pUC19) control strain, whereas the presence of the *orf14_bIL67_* gene allowed continued cell growth under the same conditions (Figure 3C).

To confirm that the improvement of cellular growth was due to the activity of the Orf14_bIL67_ protein, we performed a complementation test using the mutated *orf14_bIL67_* genes [29]. The *orf14_bIL67_*/D51A gene codes for a protein somewhat affected in its ssDNA-binding activity, this construct improved the growth of KLC789 at 38°C, albeit to a lesser extent than the wild type gene (Figure 3C). In contrast, the presence of pUC19:*orf14_bIL67_/*Y70A, coding for a mutant protein unable to bind ssDNA, did not positively affect the growth of *E. coli* KLC789 cells in non-permissive conditions (Figure 3C).

To support these results we assessed the affinity of the purified Orf14_bIL67_ protein for ssDNA at 30°C and 38°C. We used electrophoretic mobility-shift assays with a radioactively labelled single-stranded 80-mer oligonucleotide of random sequence (Materials and Methods). There was no significant difference in the ssDNA-binding pattern of Orf14_bIL67_ at the two temperatures tested (Figure 3D). This indicates that the Orf14_bIL67_ protein binds to ssDNA with a similar affinity at 30°C and 38°C. Therefore, the ability of strain KLC789 (pUC19:*orf14_bIL67_*) to form only pinpoint colonies at 38°C and partial improvement of KLC789 cell growth by *orf14_bIL67_* at this temperature is not crucially due to temperature sensitivity of Orf14_bIL67_ ssDNA-binding activity.

Thus, the phage bIL67 *orf14_bIL67_* gene is able to complement the conditional ts mutation in the strain KLC789, *E. coli ssb-1* at 38°C. This complementation was not observed with ssDNA-binding deficient mutant, indicating that it required the ssDNA-binding activity of the Orf14_bIL67_ protein.

## Discussion

SSB proteins are conserved throughout all kingdoms of life. It was suggested that prokaryotic, archaeal and eukaryotic SSBs have all descended from a common ancestor polypeptide with a single OB-fold *via* gene duplication and recombination events [11], [16], [37], [38]. Nevertheless, bacterial SSB proteins, like other proteins involved in DNA replication, are clearly different from those found in Archaea and Eukaryotes [39]–[41]. Phylogenetic trees built previously with limited numbers of ssDNA-binding proteins show two essential features of SSB superfamily : i) bacterial and mitochondrial SSBs are separated from the archaeal SSBs/eukaryotic RPAs, ii) phage and bacterial SSBs do not form monophyletic groups and are intermixed with each other [27], [33], [42]. Thus, phylogenetic analysis supports the hypothesis that the ssDNA-binding protein superfamily was derived from a common ancestral domain present in the genome of an organism at the very base of the evolutionary tree, while phage *ssb* genes were transferred from the genomes of their hosts [7], [8], [27].

**Figure 4:**
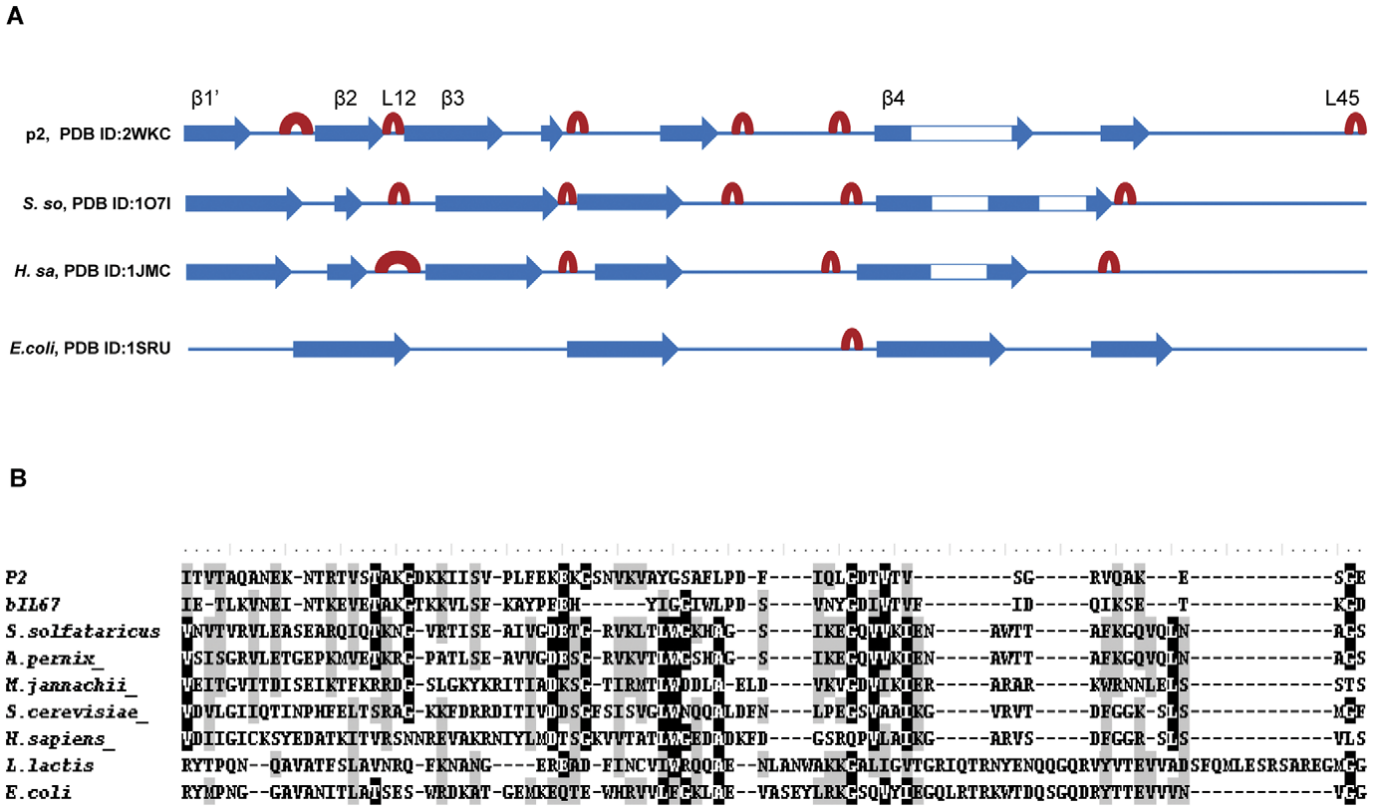
Alignment of ssDNA-binding OB-fold domains of Orf14_bIL67_–like, prokaryotic, archaeal and eukaryotic proteins. **A:** Important elements of the secondary structure of phage p2, *S. solfataricus*, *H.sapiens* and *E. coli* ssDNA-binding proteins. strands are colored in blue, loops are colored in red. Structural data are available in Protein Data Bank (PDB identifiers for p2, *S. solfataricus*, *H.sapiens* and *E. coli* ssDNA-binding proteins are 2WKC, 1O7I, 1JMC and 1SRU respectively). **B:** Bacteriophage sequences (accession numbers: AAR14301 for phage p2 SSB and AAA74351 for phage bIL67 SSB) aligned with the ssDNA-binding OB-fold domains of *S. solfataricus* SSB (accession number: AAK42515.1), *A. pernix* SSB (accession number: BAA80315), *M. jannachii* RPA (accession number: MJ1159), *E. coli* SSB (accession number: AAC43153), *L. lactis* SSB (accession number: AAK06288), *S. cerevisiae* RPA70 (accession number: CAA42420.1), *H. sapiens* RPA70 (accession number: AAS94324.1). Conserved residues are shaded grey and identical residues are shaded black.

Here we demonstrate for the first time that SSBs encoded by the well-defined group of lactococcal phages form a clearly isolated protein family. Phylogenetic analyses of the OB-fold domains distinguished the Orf14_bIL67_ protein family as a distinct clade of the domain tree, in remarkable contrast to those of other phage SSBs. The position of the Orf14_bIL67_ branch in the phylogenetic tree is consistent with a particular ssDNA-binding OB-fold domain of these proteins which shares some features with both archaeal and bacterial OB-folds. We performed multiple sequence alignment of OB-fold domains of Orf14_bIL67_ and Orf34_p2_ proteins with the domains of representative bacterial, archaeal and eukaryotic ssDNA-binding proteins using tree different methods (Materials and Methods). The sequences were aligned on the basis of sequence similarities detected previously between archaeal and eukaryotic OB-folds [19]. All methods revealed that at the sequence level phage proteins are more similar to archaeal SSBs than to bacterial SSBs. This correspond to the structural similarity between phage and archaeal OB-folds (Figure 4A, B). Within conserved or similar residues between at least one phage and archaeal SSBs, several have been proposed to play a role in ssDNA binding of the phage Orf34_p2_ SSB: R15, K21, K24 and K68 [31]. The conserved V17 residue has been shown to be involved in ssDNA binding of the phage Orf14_bIL67_ SSB [29].

Thus, although we cannot point explicitly to the origin of Orf14_bIL67_-like SSBs and their propagation pathways, some elements of the evolutionary history of Orf14_bIL67_-like SSBs can be reasonably suggested. The largest group of Orf14_bIL67_ SSB proteins is formed by proteins encoded by virulent lactococcal phages of the 936 species suggesting that the *ssb* gene was initially acquired by a 936-like phage or its ancestor. The presence of *Orf14*_bIL67_ *ssb* genes in the genomes of temperate lactococcal phages is not surprising, as recombination events causing gene transfer play important role in the evolution of both temperate and virulent lactococcal phages [43]–[45]. The restricted distribution of *Orf14_bIL67_ ssb* genes in P335-like phage genomes suggests a more recent acquisition *via* horizontal gene transfer from virulent phages. This is in agreement with the commonly accepted view that recent horizontal gene transfers tend to involve closely related organisms and ancient events involved transfer between more distant organisms [46]–[48]. A similar scenario cannot be proposed with confidence for *ssb* genes of c2-like phages because the appropriate amount of sequence data is not currently available. Homologous recombination between 936- and c2-like virulent phages is not expected to be frequent, although these phages are able to infect the same host. Nevertheless, the presence in the phylogenetic tree of the SSBs encoded by two c2-like phages may suggest a gene transfer between 936-and c2-like phages.

Here we show that unlike other phages, the virulent lactococcal phages carry *ssb* genes that were not acquired from their bacterial hosts. The emergence of the phage SSBs among the archaeal ones suggests an archaeal origin of their *ssb* gene. This indicates a unique case of horizontal gene transfer between Archaea and virulent bacterial phages. The coexistence between diverse archaeal and lactic acid bacteria populations in different types of fermentation processes is well documented [49]–[51]. This could facilitate some gene exchange between these organisms, their viruses and phages. Although phages are known to play a key role in horizontal gene transfer in Bacteria, until now there has been no evidence of their participation in gene transfer between Bacteria and Archaea and any bacterial phage was shown to infect Archaea [52].

The precise role of the Orf14_bIL67_-like SSBs in phage development is not yet established. However, it was stressed that the *ssb* genes are adjacent to the genes encoding the single-strand annealing proteins and most likely involved in phage-promoted DNA recombination [29], [31]. Elucidation of the role of these proteins in phage development is currently in progress. Here we confirmed the biological functionality of the Orf14_bIL67_ protein *in vivo* by complementation of the *E. coli ssb-1* mutant. Over-expression of highly homologous SSB proteins from different bacteria and coli-phage P1 and distinct SSB protein from *S. solfataricus* can complement the lethal phenotype of the *ssb-1* mutation [19], [25], [53], [54]. The crenarchaeal and *E. coli* proteins show moderate similarity over the N-terminal part, but share a flexible C-terminal tail involved in protein-protein interaction [11], [19]. For Orf14_bIL67_, specific complementation was not anticipated, due to amino acid sequence differences from *Eco*SSB, and the divergent structural characteristics including important differences in the C-terminal part of the proteins [29], [31]. These features were expected to assure nonspecific binding of phage SSB to ssDNA, but prevent Orf14_bIL67_ from specific protein-protein interactions important for different aspects of DNA metabolism. Indeed, the growth of *E. coli ssb-1* (ts) KLC789 at a non-permissive temperature was improved by expression of Orf14_bIL67_, but not entirely restored. This suggests that the phage bIL67 SSB protein is functional in *E. coli in vivo*, but it is most probably unable to fully fulfill the varied functions of the *Eco*SSB protein. It would be also interesting to investigate the functional similarities between Orf14_bIL67_-like SSBs and their archaeal counterparts.

The results presented here explore the unique features of the Orf14_bIL67_-like SSB proteins and their differences from other phage and bacterial SSBs. It strongly supports the hypothesis that the Orf14_bIL67_-like SSBs of lactococcal phages constitute a new protein family within the SSB superfamily. These proteins are encoded by genes which according to presented phylogeny-based analysis were acquired from archaeal origin and adapted to phage multiplication in the bacterial host. This indicates that bacterial phages can also contribute to the horizontal gene transfer between Bacteria and Archaea.

## Materials and Methods

### Data collection, phylogenetic and sequence analysis

SSB sequences were retrieved from GenBank (http://www.ncbi.nlm.nih.gov) by running BLAST searches using reference SSB sequences from Bacteria, Archaea, Eukaryotes and phages (AAC43153 for *E.coli* SSB; NP_391512 for *B. subtilis*, AAK06288 for *L. lactis*, AAK42515 for *S. solfataricus* SSB; MJ1159 for *Methanococcus jannachii* RPA; 6319321 for *Saccharomyces cerevisiae* RPA70; 1350579 for *Homo sapiens* RPA70; AAQ14098 for phage P1 SSB, NP_041970 for phage T7 gp2.5, AAR14301 for phage p2 SSB and AAA74351 for phage bIL67 SSB). The resulting SSB dataset included 729 sequences (Table S1). To build the OB-fold SSB dataset all sequences were trimmed to contain only the OB-fold domain. Homology searches were carried out using the PSI-BLAST program [55]. Sequences alignment was performed on 78 sequences sharing no more than 85% identity while preserving the original balance between the 10 protein subfamilies. Sequences were aligned with Clustal X 2.0 (default settings) and MUSCLE (default settings) and then manually refined using Bioedit V7 [56]–[58]. To account for protein structure, sequences were aligned using the “accurate” mode of T-coffee (Figure S5). This mode searches each sequence against the PDB and when a high quality match is found uses structure information instead of just sequence information to align sequences [59]. Multiple sequence alignments of OB-fold domains of Orf14_bIL67_ and Orf34_p2_ proteins with the domains of representative bacterial (*E. coli*, *L. lactis*), archaeal (*S. solfataricus A. pernix*, *M. jannachii*) and eukaryotic (*S. cerevisiae*, *H. sapiens*) ssDNA-binding proteins were performed using alignment software ClustalX, Muscle and Mafft available in Bioinformatics platform of Max Planck Institute for Developmental Biology (http://toolkit.tuebingen.mpg.de/sections/alignment).

Cluster Analysis of Sequences (CLANS) was used to identify subfamilies of closely related SSB sequences and elucidate the relationships between and within the ssDNA-binding protein subfamilies [32]. CLANS is a Java utility based on the Fruchterman-Reingold graph layout algorithm. It runs BLAST on given sequences, all-against-all, and clusters them in 3D according to their similarity. A 2D-representation was obtained by seeding sequences randomly in the arbitrary distance space.

Accession numbers of p2, bIL66M1, sk1, bIL170, jj50, P008, bIBB29, 712, c2, bIL67, P335, SL4, SB13, SB14, CB19 and CB20 phage genomes are GQ979703, AY249139, AF011378, AF009630, DQ227764, DQ054536, EU221285, DQ227763, L48605, L33769, DQ838728 and FJ848881 - FJ848885. Accession number of *Oenococcus oeni* AWRIB429 429 and prophage “2” *of L. lactis* subsp. *cremoris* SK11 are ACSE00000000.1 (contig 50, accession number ACSE01000050.1) and CP000425.

We employed both Neighbour Joining (NJ) and Maximum Likelihood (ML) methods in our phylogenetic analyses. MEGA V4 was used for NJ analyses with 100 bootstrap replicates [60]. PhyML v3.0 was used for ML analyses with 200 bootstrap replicates [61]. For ML and NJ analyses, we used a smaller alignment made of 78 OB-fold domains from a representative sample of the 10 subfamilies identified by CLANS. The sequences were chosen so that two sequences have no more than 85% identity. We then selected a model of sequence evolution (LG+G+F) from aligned sequences using ProtTest V2.4 [62]. Rather than using the default settings (BIONJ starting tree, discrete gamma with four rate categories, NNI moves) and following the guidelines in the operating manual for PhyML v3.0, we used SPR moves with ten starting trees for a more thorough exploration of the tree space (options –alpha e –search BEST –rand_start –n_rand_starts 10 of PhyML).

### Bacterial strains and growth conditions

The *E. coli* strains used in study were TG1 [*sup*E Δ(*hsd*M-*mcr*B) (*r_k_^−^m_k_^−^Mcr*B^−^) *thi* Δ *lac-pro*AB] F′(*tra*D36 *lac*I^q^ *lac*Z ΔM15 *pro*A^+^B^+^) and the thermosensitive *ssb-1* KLC789 mutant (F^−^, *metA7*, *rha8*, *thyA36*, *amp50*, *deoC2*, *ssb-1*) [36]. Strains were grown on LB medium: - strain TG1 at 37°C, and strain KLC789 at 30°C or 38°C. When necessary, ampicillin (100 µg ml^−1^), IPTG (0.25 or 0.5 mM) and X-gal (50 µg ml^−1^) were added to the medium.

### Molecular manipulations and DNA sequencing

DNA manipulations, cloning procedures and transformation of *E. coli* cells were performed as described elsewhere [63]. Digestions with restriction enzymes (New England Biolabs) were done as recommended by the supplier. Cycle extension reactions were used for DNA sequencing with specific primers, Taq polymerase and fluorescent dye-coupled dideoxynucleotides (Applied Biosystems) on a 370A DNA Sequencer (Applied Biosystems).

### *In vivo* complementation

The *orf14_bIL67_* wild and mutant genes that encode Orf14_bIL67_/Y70A and Orf14_bIL67_/D51A were amplified with oligonucleotides: 5′-C**GGGATCC**TTAGAATGGTAATG- 3′, 5′-GC**TCTAGA**AATAATTTTGTTTAAC-3′ using as template pTYB recombinant plasmids constructed previously [29]. BamHI and XbaI restriction sites, indicated in bold, were used to insert the PCR products into pUC19 (New England Biolabs). The resulting constructs were used to transform *E. coli* TG1 cells. After confirming the sequence of the inserted DNA fragments, the recombinant plasmids were introduced into strain KLC789 carrying the *ssb-1* allele. Ten independent clones of each KLC789 (pUC19), KLC789 (pUC19:*orf14_bIL67_*), KLC789 (pUC19:*orf14_bIL67_*/D51A) and KLC789 (pUC19:*orf14_bIL67_*/Y70A) were grown in 500 µl of LB supplemented with ampicillin at 30°C or 38°C in a Bioscreen C apparatus (LabSystems), with continuous monitoring of OD_600_. For plating experiment *E. coli* KLC789 cells were transformed with pUC19 or pUC19:*orf14_bIL67_* plasmid DNA as described elsewhere [63]. Single transformant of each strain was than propagated in LB broth at 30°C for 6 hours, adequate dilutions were plated onto the LB agar plates in the presence of 0.25 mM of IPTG and incubated at 30°C for 18 hours.

### Electrophoretic mobility-shift assay (EMSA)

Orf14_bIL67_ protein was overproduced and purified using the IMPACT-CN purification system (New England Biolabs) as described previously [29]. Binding of the Orf14_bIL67_ to DNA was evaluated by electrophoretic mobility-shift assays (EMSA) with an 80-mer oligonucleotide (Genosys, Sigma): 5′-TTTGTCGGTACTTTATATTCTCTTATTACTGGCTCGAAAATGCCTCTGCCTAAATTACATGTTGGCGTTGTTAAATATGGGGG - 3′ (random φM13-derived sequence) with a ^32^P-γdATP (3000 Ci mmol^−1^) labelled 5′. Binding reactions (20 µl) were carried out in binding buffer (20 mM Tris-HCl, 25 mM KCl, 1 mM EDTA, 1 mM DTT, 2.5% glycerol, 0.3 mg ml^−1^ BSA, pH 7.5) by mixing a fixed amount (0.5 nM) of the radioactively labelled oligonucleotide with each of a series of concentrations of the protein (up to 100 nM). The samples were incubated for 10 min at 30°C or 38°C and run on a non-denaturing 5% polyacrylamide gel ran for 2 hours at 200 V in 0.25×TBE buffer at 4°C. The gel was dried under vacuum and the binding pattern was determined by autoradiography using a PhosphoImager.

## Supporting Information

Figure S1 **Gene context analysis of the DNA fragments in** ***O. oeni*** **AWRIB429 429 genome shotgun sequence.** The conserved gene clusters in *Oenococcus oeni* AWRIB429 429 and lactococcal phage genomes were detected using Gene Context Analysis in the Integrated Microbial Genomes (IMG) data management system (http://img.jgi.doe.gov/). Five upper lines represent *O. oeni* AWRIB429 429 contigs from whole genome shortgun sequence. Four lower lines represent early genome regions from representative *L. lactis* 936-like phages. Genes encoding homologous proteins (from 77% to 98% aa identity) are marked by the same color. Genes encoding Orf14_bIL67_ -like SSBs proteins are shown in red. Accession numbers of *O. oeni* and phage sequences are indicated above each line.
(TIF)

Figure S2 **Cluster map of the ssDNA-binding protein superfamily.** The complete sequence dataset for SSB proteins containing only the OB-fold domain was clustered using CLANS (Materials and Methods). A 2D representation was obtained by seeding sequences randomly in the arbitrary distance space. In the network, each dot represents a single protein. Sequences of phages encoding Orf14_bIL67_ -like SSBs proteins are shown in red. Other colours: purple – Crenarchaea , blue – Euryarchaea, maroon – Eukaryotes, dark blue – mitochondria, black – Gram-negative bacteria, green – Gram-positive bacteria.
(PDF)

Figure S3 **Unrooted Neighbour Joining (NJ) phylogenetic tree of the ssDNA-binding proteins.** The SSB phylogeny was reconstructed from OB-fold multiple alignment of 78 ssDNA-binding protein sequences that was generated using the Clustal X 2.0 (Materials and Methods). Bootstrap support values are shown. Sequences of phages encoding Orf14_bIL67_ -like SSBs proteins are shown in red. Other colours: purple – Crenarchaea , blue – Euryarchaea, maroon – Eukaryotes, dark blue – mitochondria, black – Gram-negative bacteria, green – Gram-positive bacteria, and olive – phages.
(PDF)

Figure S4 **Maximum Likelihood (ML) phylogenetic trees of the ssDNA-binding proteins.** The SSB phylogenies were reconstructed from OB-fold multiple alignments of 78 ssDNA-binding protein sequences that were generated using MUSCLE (A) and Clustal X 2.0 (B) (Materials and Methods). The trees are rooted arbitrary. Bootstrap support values >50% are shown. The tip labels correspond to sequence gi numbers. Sequences of phages encoding Orf14_bIL67_ -like SSBs proteins are shown in red. Other colours: purple – Crenarchaea , blue – Euryarchaea, maroon – Eukaryotes, dark blue – mitochondria, black – Gram-negative bacteria, green – Gram-positive bacteria, and olive – phages. Branches differing between each tree and the tree represented in Fig. 2 are colored in gray.
(PDF)

Figure S5 **OB-fold multiple sequence alignment.** A representative and balanced sample of 78 ssDNA-binding protein sequences from the 10 identified families were aligned with “accurate” mode of T-coffee [59]. Boxes are color-coded according to the chemical nature of the conserved amino acid residues.
(PNG)

Table S1 **Data set of the ssDNA-binding proteins.** SSB sequences were retrieved from GenBank (http://www.ncbi.nlm.nih.gov/) by running BLAST searches using reference SSB sequences from Bacteria, Archaea, Eukaryotes and phages (Materials and Methods). The resulting SSB dataset included 729 sequences.
(XLSX)
