# Supplementary figures and images for: Phylogenetic and Complementation Analysis of a Single-Stranded DNA Binding Protein Family from Lactococcal Phages Indicates a Non-Bacterial Origin
Source: PLoS One. 2011 Nov 4;6(11):e26942. doi: 10.1371/journal.pone.0026942 (PMC3208561; doi:10.1371/journal.pone.0026942)

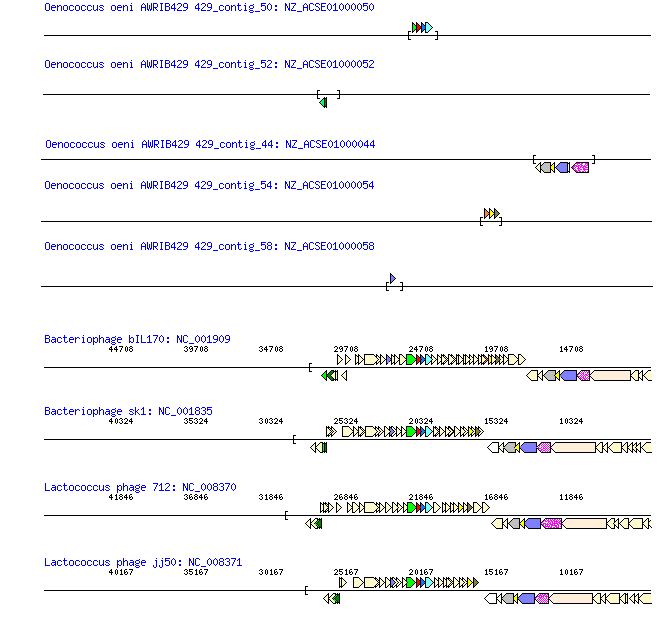

Supplement: Figure S1 — Gene context analysis of the DNA fragments in O. oeni AWRIB429 429 genome shotgun sequence. The conserved gene clusters in Oenococcus oeni AWRIB429 429 and lactococcal phage genomes were detected using Gene Context Analysis in the Integrated Microbial Genomes (IMG) data management system (http://img.jgi.doe.gov/). Five upper lines represent O. oeni AWRIB429 429 contigs from whole genome shortgun sequence. Four lower lines represent early genome regions from representative L. lactis 936-like phages. Genes encoding homologous proteins (from 77% to 98% aa identity) are marked by the same color. Genes encoding Orf14bIL67 -like SSBs proteins are shown in red. Accession numbers of O. oeni and phage sequences are indicated above each line. (TIF) [file pone.0026942.s001.tif]

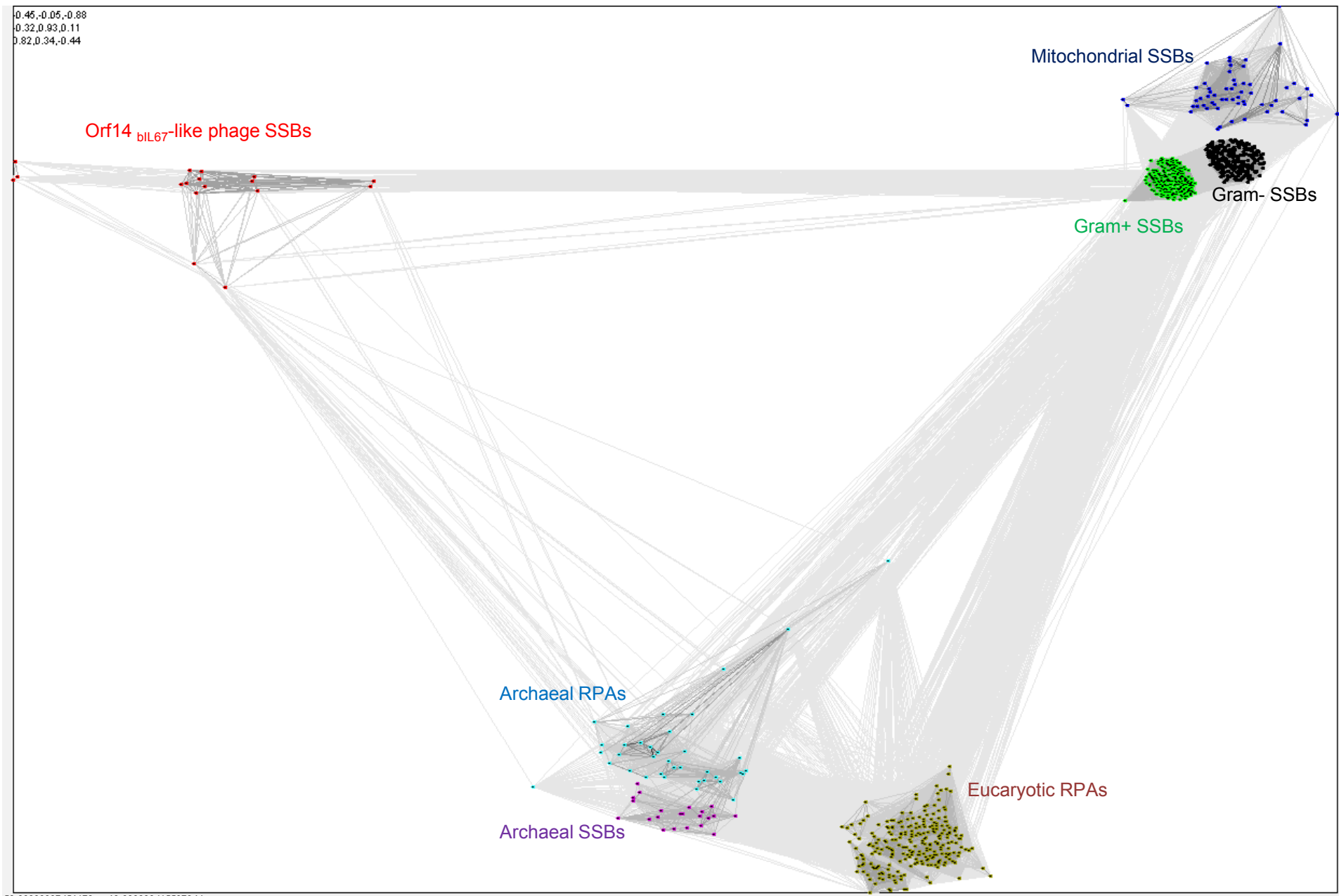

Supplement: Figure S2 — Cluster map of the ssDNA-binding protein superfamily. The complete sequence dataset for SSB proteins containing only the OB-fold domain was clustered using CLANS (Materials and Methods). A 2D representation was obtained by seeding sequences randomly in the arbitrary distance space. In the network, each dot represents a single protein. Sequences of phages encoding Orf14bIL67 -like SSBs proteins are shown in red. Other colours: purple – Crenarchaea , blue – Euryarchaea, maroon – Eukaryotes, dark blue – mitochondria, black – Gram-negative bacteria, green – Gram-positive bacteria. (PDF) [file pone.0026942.s002.pdf]

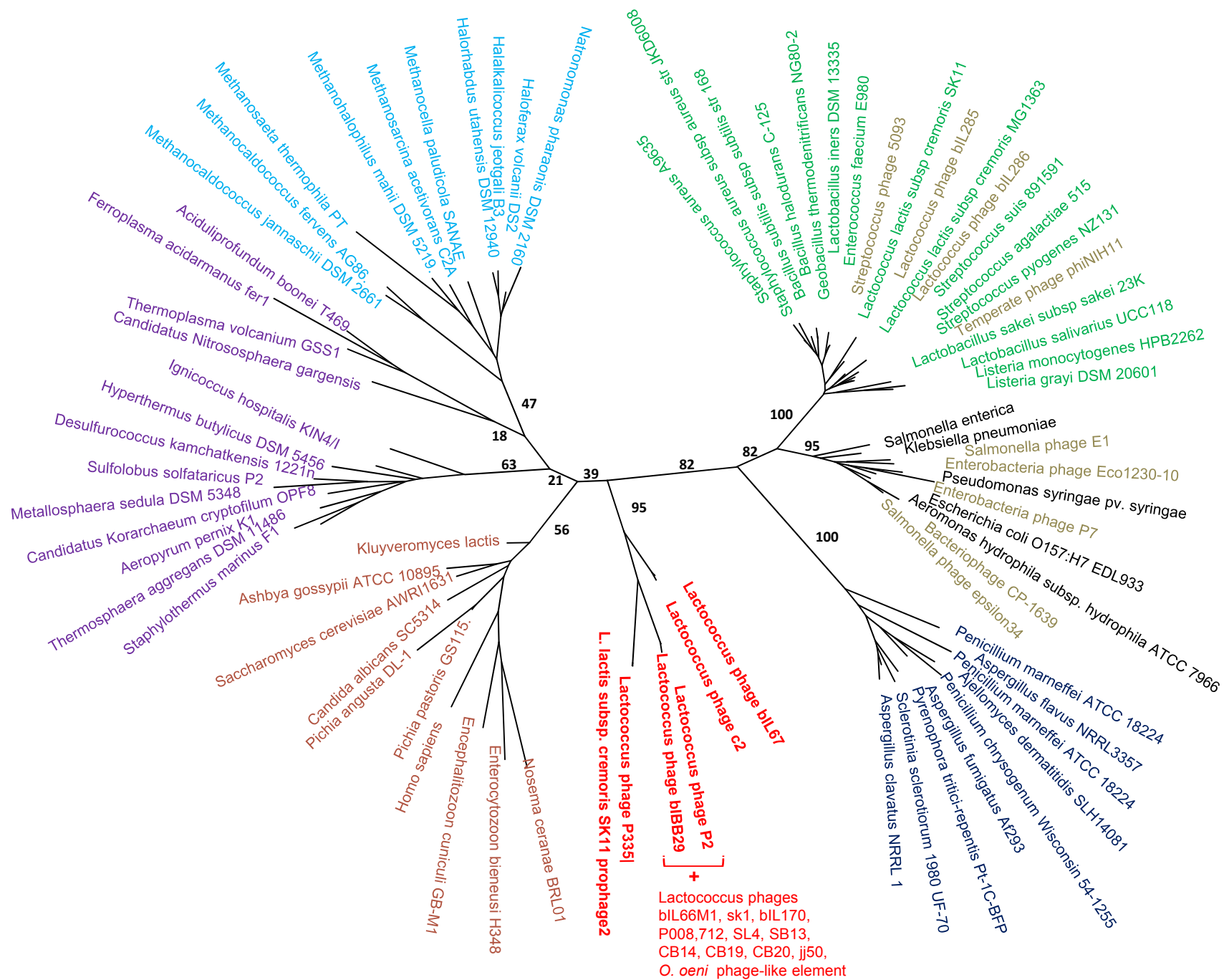

Supplement: Figure S3 — Unrooted Neighbour Joining (NJ) phylogenetic tree of the ssDNA-binding proteins. The SSB phylogeny was reconstructed from OB-fold multiple alignment of 78 ssDNA-binding protein sequences that was generated using the Clustal X 2.0 (Materials and Methods). Bootstrap support values are shown. Sequences of phages encoding Orf14bIL67 -like SSBs proteins are shown in red. Other colours: purple – Crenarchaea , blue – Euryarchaea, maroon – Eukaryotes, dark blue – mitochondria, black – Gram-negative bacteria, green – Gram-positive bacteria, and olive – phages. (PDF) [file pone.0026942.s003.pdf]

A

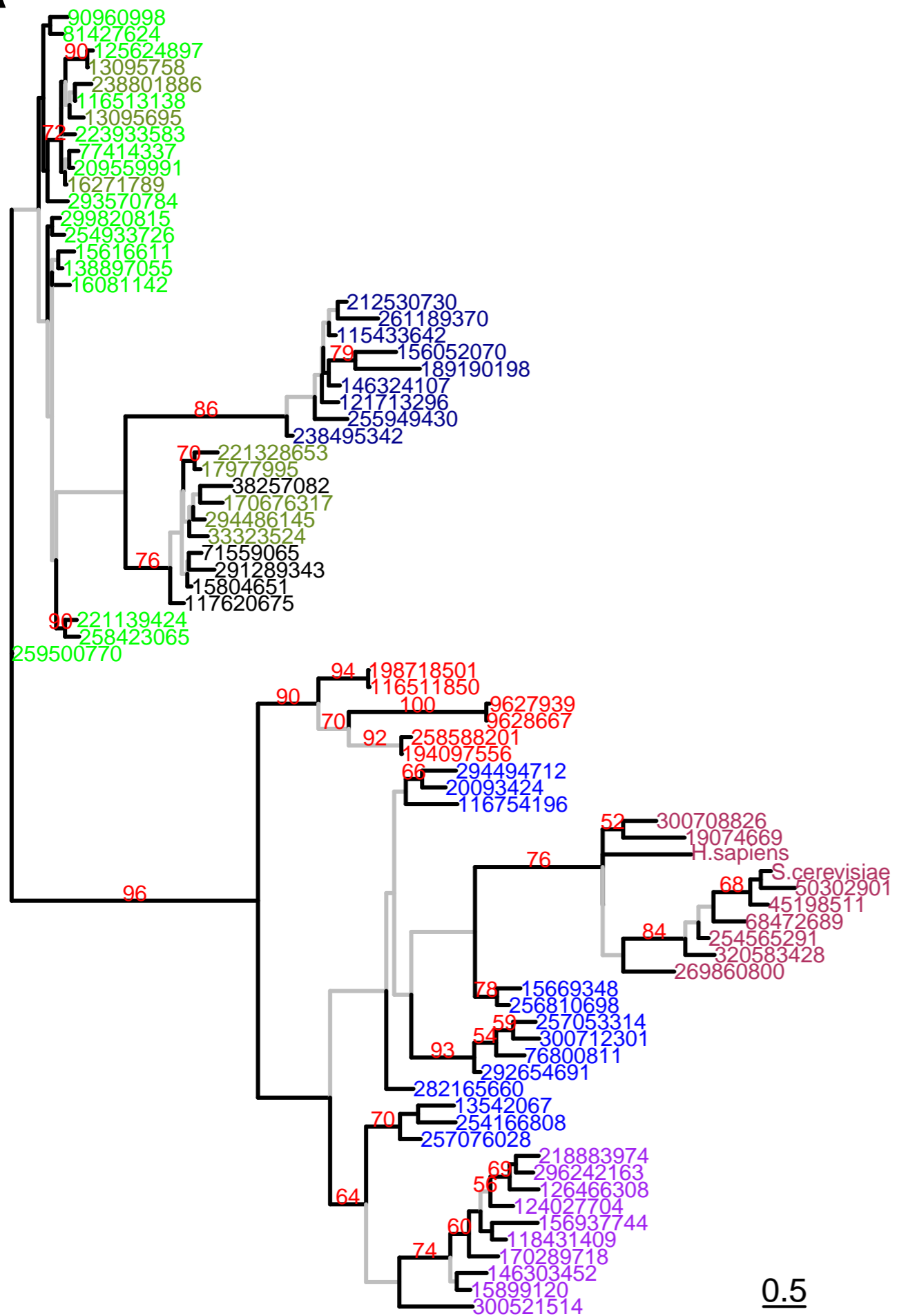

B

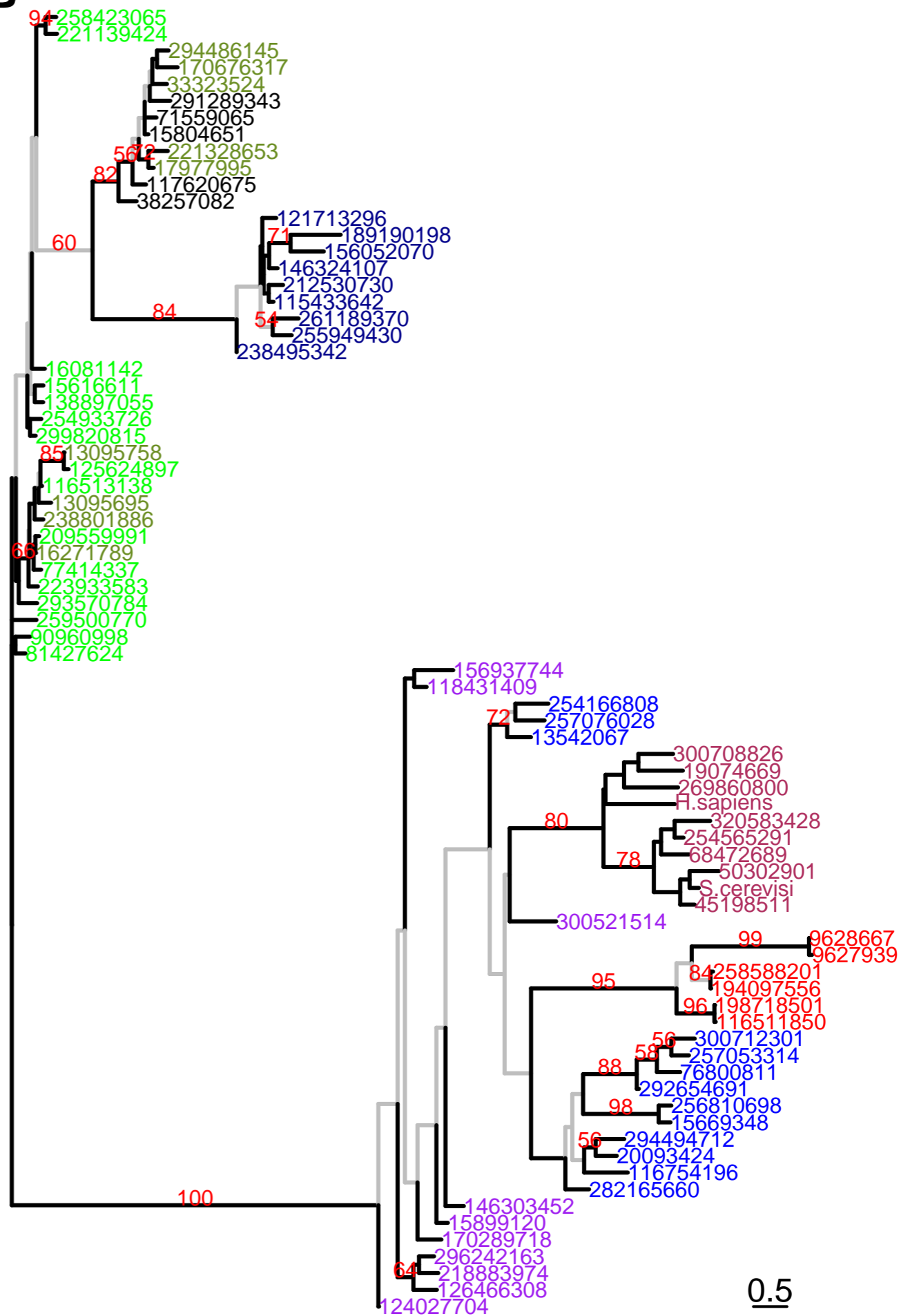

Supplement: Figure S4 — Maximum Likelihood (ML) phylogenetic trees of the ssDNA-binding proteins. The SSB phylogenies were reconstructed from OB-fold multiple alignments of 78 ssDNA-binding protein sequences that were generated using MUSCLE (A) and Clustal X 2.0 (B) (Materials and Methods). The trees are rooted arbitrary. Bootstrap support values >50% are shown. The tip labels correspond to sequence gi numbers. Sequences of phages encoding Orf14bIL67 -like SSBs proteins are shown in red. Other colours: purple – Crenarchaea , blue – Euryarchaea, maroon – Eukaryotes, dark blue – mitochondria, black – Gram-negative bacteria, green – Gram-positive bacteria, and olive – phages. Branches differing between each tree and the tree represented in Fig. 2 are colored in gray. (PDF) [file pone.0026942.s004.pdf]

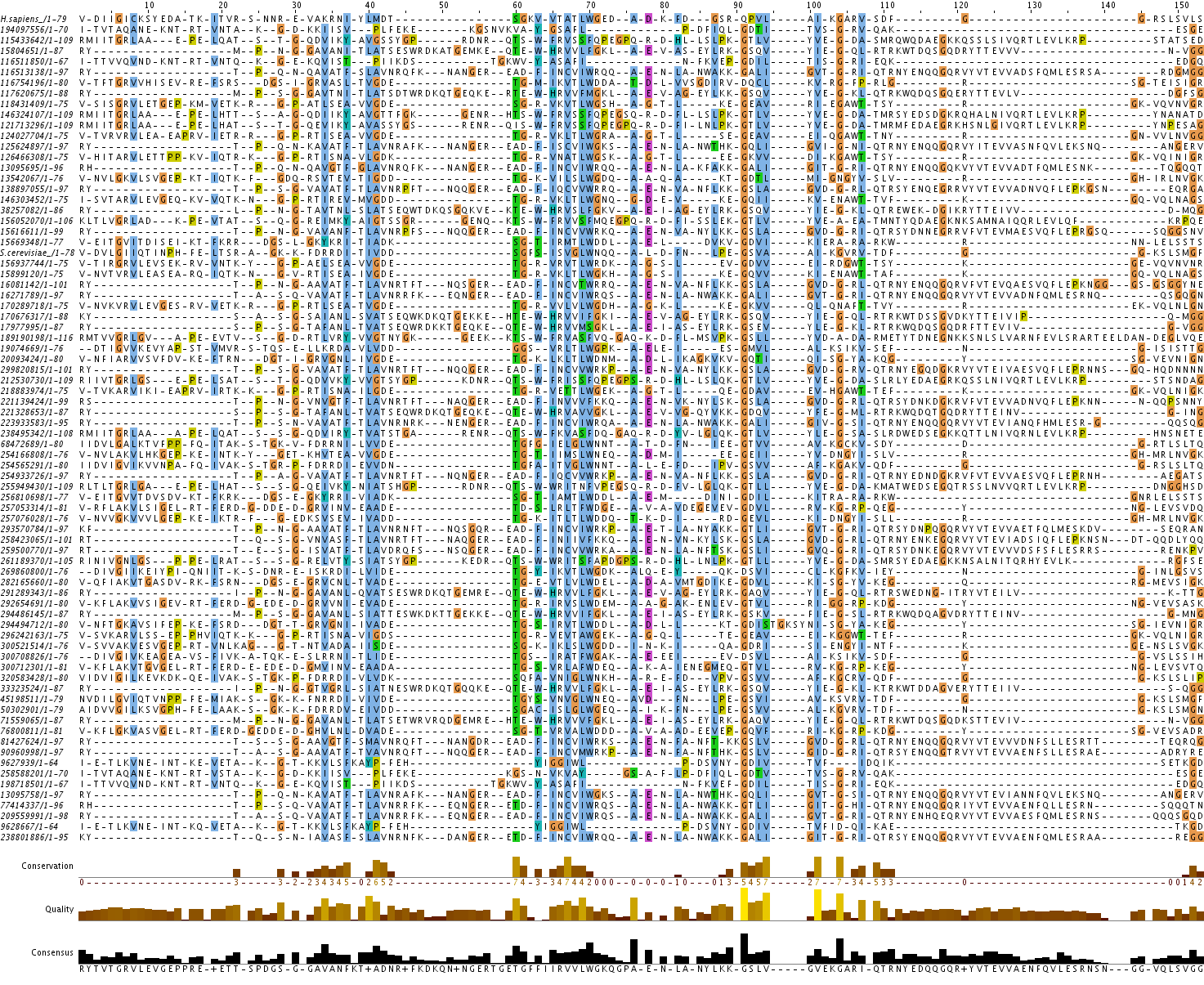

Supplement: Figure S5 — OB-fold multiple sequence alignment. A representative and balanced sample of 78 ssDNA-binding protein sequences from the 10 identified families were aligned with “accurate” mode of T-coffee [59]. Boxes are color-coded according to the chemical nature of the conserved amino acid residues. (PNG) [file pone.0026942.s005.png]
